# The influence of intrinsic and extrinsic motivational factors on e-WOM behaviour: The role of psychological impact during the time of COVID-19 crisis

**DOI:** 10.1016/j.heliyon.2023.e13270

**Published:** 2023-01-29

**Authors:** Balamoorthy S, Bibhas Chandra

**Affiliations:** Department of Management Studies & Industrial Engineering, Indian Institute of Technology (ISM), Dhanbad, 826004, Jharkhand, India

**Keywords:** Social mobile app, COVID-19, e-WOM, Personality, Intrinsic-extrinsic motivation, Psychological impact

## Abstract

This investigation unearths the trait antecedents of intrinsic and extrinsic motivational drivers and their consequences on attitude towards e-WOM of social mobile app users. Additionally, this study attempts to explore the moderating role of psychological impact on attitude towards e-WOM when COVID-19 is ravaging the world. Following the descriptive research method, the survey was carried out among convenience sample respondents of 627 social mobile app users. The outcome brought out that extraversion had a favourable influence on opinion leadership, customer engagement, and self-expression for sharing consumption related information during the pandemic. Likewise, materialism also had a strong effect on self-expression. Nonetheless, extraversion and materialism failed to affect self-presentation motives. And, self-presentation was observed influencing attitude towards e-WOM negatively. The outcome of the study demonstrates that individuals with high psychological impact have a strong motivation to involve in customer engagement to participate in e-WOM communication. Motivational drivers such as self-expression, opinion leadership, and customer engagement had a significant effect on attitude towards e-WOM for the whole sample analysis. This investigation contributes to existing literature of e-WOM behaviour and widens the scope of self-presentation theory, social exchange theory, theory of planned behaviour etc. Finally, this research also assists marketers and practitioners to take better decision-making on developing a social media advertising and campaign using the framework.

## 1 Introduction

Social mobile apps or Mobile social networks (MSNs) are facilitated by the combination of social and mobile computing [1]. It has features similar to that of social networking sites (SNS) from online social websites like Web 2.0 technologies and online communities, which can be accessed through smartphones [2]. The social mobile app provides ample opportunities for marketers as the mobile internet user base is expected to cross over 600 million in 2022 with 450 million social media users making India a mobile-first market [3]. Further, surpassing 27 billion downloads of mobile apps, India became the second largest mobile app downloader; these users also spend about 4.7 h per day on average with mobile apps, where 70.8% of this time is spent exclusively on social media apps [4]. This information clearly implies that social mobile apps play a pivotal role in individuals' day-to-day life [5]. This has enormously changed the way how people interact and socialise with each other. There may be several reasons for this change. Due to its unique physical architecture, users' self-presentation unfolds distinctly across different social media [6]. These platforms offer opportunities for self-expression and possibly contributes to the self-presentation of users [7]. Indeed, it is clear that social media helps to attain self-presentational goals through posting photographs and updating profile [8]. As a result, media features and intrinsic-extrinsic factors affect an individual's self-presentation on SNS [9]. However, people show varying intensity while posting their personal and consumption-related experiences on social media [10]. Like, some of them share too often and some rarely because e-WOM is individualistic to an extent and a disjointed kind of engagement exists on social media.

The undulating motivations to express oneself may have some underlying causes, which have been researched diligently in the past and reported in many studies, like “why people engage in e-WOM” [[11], [12], [13], [14]]. In addition, Huang [15] attributed information sharing behaviour to personality traits, which are associated with social media status updates and self-presentation strategies. Specifically, personality traits are related to behaviour on social media [16,17]. Thus, social media naturally appeals to extraverts as they have a desire for social attention [18] and social interaction [19]. More importantly, extraversion is the strongest predictor of SNS use [20] and is also favourably related to the use of the internet for leisure services and sharing information [21]. And, extraversion is stated to be spending more time on social media creating content [22]. Supporting this, a stream studies have been undertaken to understand the effect of extraversion on selfie-posting [23,24], and self-disclosure [25] on social media. Further, materialistic individuals display their status, wealth and uniqueness in order to impress others [26] by using products to disclose information about themselves [27]. Thus, materialism had an impact on the status perception of individuals on SNS [28]. Besides, recent research on trait materialism has also shown the influence on posting behaviour [29], e-WOM diffusion [30], and SNS intensity among users [31].

Apart from personality traits, intrinsic and extrinsic rewards (motivational drivers) also played a crucial role in writing reviews online [32], e-WOM intention [11], sharing behaviour on social media [33]. Considering motivational theory, Teichmann et al. [34], found that extrinsic rewards such as self-presentation and opinion leadership had a strong effect on creating content online. Similarly, social media users’ self-presentation has been observed in past studies [[35], [36], [37], [38]]. To illustrate, self-presentation on SNS is connected to social connection [39], the need to belong [40], sharing brand-related content [41], likes and comments [42,43] and e-WOM [44]. Relatedly, a host of studies also unearthed the drivers of self-presentation [[45], [46], [47], [48]]. Furthermore, extant studies explored the drivers and consequences of opinion leadership using an integrated approach [[49], [50], [51], [52]]. Exclusively, research had also been undertaken in the past to probe the effect of opinion leadership on interaction intention [53] and e-WOM intention [11]. This line of research suggests that opinion leadership is a key element in the e-WOM behaviour of individuals on social media.

Intrinsic motivational drivers such as customer engagement and self-expression contributes to consumer-generated content on social media enormously. Cheung et al. [54], showed that social media is a better way to enhance customer engagement and also proposed ways to improve customer engagement. Implications and significance of customer engagement were highlighted by researchers in the marketing literature widely in the past [[55], [56], [57], [58], [59]]. Moreover, scholars have also documented the antecedents of customer engagement [55,[60], [61], [62]]. Further, self-expression is the process of conveying to others who they are. This can also be achieved by consuming some products or brands which may be perceived as potential for expressing one's self [63]. Briefly, it is viewed here as a motive for self-expression to build their own image through consumption experience and interaction with others. Applying this, Morgan and Townsend [64] unearthed how hedonic and utilitarian benefit drive self-expression through consumption. Consequently, self-expression had a positive influence on consumer choice [63], and purchase motivation [65]. Likewise, self-expression had also a major effect on social capital and bridging social capital [66], content creation [67], and e-WOM [12]. Evidence of support shown that how self-expression is a major component in determining the online behaviour of individuals. In short, authors stress the importance to recognise the extent to which intrinsic-extrinsic motivational drivers play a vital role in appreciating the e-WOM behaviour in comprehensive approach.

The personality traits and their effect on e-WOM behaviour have been increasingly getting scholarly attention; yet the debate continues that whether these traits are sufficient enough to gauge human behaviour or not [68,69], and attributing personality traits to technology context is inconclusive [70]. Later, Yu and Kim [71] stated that psychological aspect of SNS users should be included to explore e-WOM behaviour. Plus, Nguyen [72] also asserted the requirement of further research to explore the influence of materialism on content generated by SNS users. Adding further, there is also a growing need to analyse the ability of personality traits to predict sharing behaviour on SNS platforms [30,69,73]. Scholars also urged additional investigations on intrinsic and extrinsic motivational drivers on creating content [34,74] and writing behaviour online [32,75]. Hence, further research is inevitable and warranted in examining the role of personality traits, intrinsic and extrinsic motivational drivers in influencing e-WOM behaviour [11].

Overall, past research highlighted that most of the studies so far have analysed the effect of personality traits, and intrinsic-extrinsic motivational drivers on e-WOM intention individually. Nevertheless, the studies involving a comprehensive approach considering these factors in combination, remain sparse and unattended. A deeper understanding of the consequences of personality traits on e-WOM behaviour on social media is still needed. Therefore, the central purpose of this investigation is to address the lack of personality traits research in exploring the impact of intrinsic and extrinsic motives on e-WOM behaviour of social mobile app users, adopting psychological impact as moderator in a comprehensive model during the time of pandemic. Hence, this investigation contributes to existing literature of consumer behaviour in widening the scope of self-presentation theory, social exchange theory, theory of planned behaviour etc. This work also expands the horizon of e-WOM domain integrating intrinsic-extrinsic motivation and personality following a comprehensive approach. Finally, this research also assists marketers and practitioners to take better decision-making on developing a social media advertising and campaign using this framework.

The remaining part of this article is structured as follows. Chapter 2 reviews the literature on personality, social exchange theory, social presentation theory, all the constructs availed, and also illustrates the hypotheses developed. Chapter 3 describes the methodology part. Chapter 4 explains data analysis and results. Chapter 5 presents discussions, contributions and implications. Chapter 6 portrays limitations and conclusions.

## 2 Literature review and theoretical support

According to social exchange theory (SET), individuals incline to provide valuable things for relationships to develop mutual beneﬁts [76]. Its main emphasis involves intangible benefits like interaction, social services, relationships, and tangible benefits such as money, rewards etc. [77]. Applying this, several studies have been carried out underpinning social exchange theory in the recent past [[78], [79], [80]]. Further, Goffman's [81] self-presentation theory holds that individuals are consciously involved in expressing themselves in order to reach the predetermined goal to impress others. This theory also stressed the importance of status and acceptance for individuals to live in a group, making self-presentation crucial to human life. Applying this, previous studies have also embraced self-presentation as an impression [[82], [83], [84]], and an important driver of e-WOM and sharing behaviour [74,85]. Moreover, intrinsic motivation is referred as “the fact of doing an activity for its own sake: the activity itself is interesting, engaging, or in some way satisfying” whereas extrinsic motivation means the “engagement in response to something apart from its own sake, such as reward or recognition or the dictates of other people” [86]. Considering Holbrook's value dimensions, Teichmann et al. [34], studied extrinsic motivations: self-presentation and opinion leadership on content creation. Adding further, this research investigates the significance of intrinsic motivational drivers such as self-expression and customer engagement on social media along the line of Algesheimer et al. [87], and Chai et al. [11], in a comprehensive model. Several studies in the past used intrinsic and extrinsic motivation concept to explore behaviour [86], knowledge sharing behaviour [88], e-WOM behaviour [11,32,33]. In order to unearth this phenomenon further, applying intrinsic-extrinsic motivation, social exchange theory and self-presentation theory [81,89] have been adopted. In the spirit of these theoretical support, the following constructs have been reviewed for this investigation.

### 2.1 Constructs

#### 2.1.1 Extraversion

Extraversion is related to energy, activity, sociability [90], social participation, and involvement with other people [91]. This trait represents people who like to get others’ attention and social interaction [19]. Given the nature of these individuals, this trait is largely associated with larger network [92]. As a result, extraversion is highly related to spending more time on social media [93], usage of SNS [20], and selfie-posting behaviour [94]. Therefore, extraversion is a vital personality trait to appreciate the e-WOM behaviour on social media in a comprehensive approach.

#### 2.1.2 Materialism

Materialism may be seen as a personal value or personality traits [95]. In Richins and Dawson's [96] work, materialism refers to “the importance one puts on acquiring and owning material possessions in one's life”. Further, they stated materialists predominantly evaluate their own success in comparison with others through the quality and quantity of their possessions. This represents how much importance individuals give to worldly possessions [97]. Briefly, this investigation views materialism as a personality trait along the line of Moisescu et al., [30]. In addition, people generate certain pleasure from their acquired product if they value materialistic goods [98]. And, they share knowledge of the brand if the product meets their expectation [99]. Therefore, materialistic individuals assume material possession is an important one to achieve in life [100]. Applying the role of materialism in social media, researches had been undertaken to elicit the e-WOM behaviour of individuals [31,98].

#### 2.1.3 Self-presentation

Generally, self-presentation is urged by individuals' desire to monitor their images in social interaction [101]. It is referred to as a highly selective and goal-directed activity whereby people control information to develop a favourable impression on audiences [102]. Following Holbrook's [103] value dimension, this investigation employs self-presentation as an extrinsic motivational driver as advocated by Teichmann et al. [34], to bring out e-WOM behaviour of social mobile app users. And, this is conceptualised as the process by which people strive to control and manage how others do perceive and judge he or she in social circumstances [104]. Based on this, a research disclosed the importance of studying self-presentation in a social context [105]. A recent study disclosed that different self-presentation strategies had impact on participation to luxury brands' advertisements on social media [106]. However, a research also observed that self-presentation is not invariably a successful way to reach favourable outcomes [107].

#### 2.1.4 Opinion leadership

Opinion leadership is recognised as an extrinsic motivational driver [34,103] and it is defined “as the extent to which individuals tend to give information or advice to others on product or service purchase decision” [108]. This emerges from a person's knowledge of a certain product category along with the intention to share information to influence others [109]. Since it is an era of social media, people found an easy way to become an opinion leaders to disseminate information. These opinion leaders usually share their knowledge on product quality, features and the experience of using it [110]. Advancing further, a study highlighted the significance of the relationship between personality and opinion leaders on SNS [111].

#### 2.1.5 Self-expression

Self-expression is often seen as intrinsic motivation [11]. It is defined “as the act of conveying one's personal experience, attitude, and identity to others when communicating with others” [112]. This implies that individuals share information about themselves to create a certain impression of the individual [113] on social media through self-expression [66]. A number of ways one can reveal self-expression through consumption in the modern marketplace [64]. Communicating through social networking sites may fulfil the desire for self-expression [114] as users engage with the brands ‘following’ and ‘liking’ the brands [115]. Due to this reason, social media makes things easier for users to meet self-expression [116]. Differently, Hollenbeck and Kaikati [117] argued that situational context had an impact on self-expression motive. Thus, it leads to a discussion on how researchers conceptualise self-expression from behavioural mechanism to motivational driver of individuals. Often, the theory of planned behaviour [118] and the uses & gratification theory [119] have been adopted to explore self-expression. It was observed that social identity theory is also commonly used to assess the association between self-expression and e-WOM [12].

#### 2.1.6 Customer engagement

Engagement is an intrinsic motivational driver [87] and “defined as the level of customers' motivational, brand-related and context-dependent state of mind characterised by specific levels of cognitive, emotional and behavioural activity in brand interactions” [120]. In the behavioural paradigm, customer engagement is a behavioural manifestation with reference to firm, brand or any purchases [121]. Further, from a practitioner's perspective, customer engagement helps in repeated interactions with the firm and facilitates the psychological, emotional and physical investment of a customer in the brand [122]. Usually, customer engagement activities are initiated by the customer [123]. This concept brings significant contributions to both practitioners and academicians in the era of digital [124]. In this way, customer engagement facilitated researchers to dig out the e-WOM behaviour in the past [125].

#### 2.1.7 Attitude towards e-WOM

Attitude towards the electronic word of mouth is a vital construct regarding social media context [126]. Generally, attitude is defined as “a learned disposition to respond towards an object in a positive or negative way” [127]. According to Fishbein and Ajzen [127] attitude is part of a belief-attitude-intention framework. Usually, attitude is classified in two ways: affective attitude and cognitive attitude [128]. Affective attitude is seen as the degree to which an individual likes an object whereas cognitive attitude is viewed as person's belief about it [128]. Thus, attitude towards e-WOM is viewed here as social mobile app users' overall assessment of e-WOM regarding consumption-related experience. Specifically “any positive or negative statement made by potential, actual, or former customers about a product or company which is made available to a multitude of people and institutions via the Internet” [129].

#### 2.1.8 e-WOM intention

e-WOM is meant for information consumers gather from interpersonal sources like friends and family [130]. Generally, e-WOM is referred to as informal communication that takes place on the internet medium irrespective of the information valence [129].In short, e-WOM is referred to as interpersonal communication that takes place among consumers with reference to a product, service or company on internet-based technology [131]. Based on this, the e-WOM intention is defined in this study as the degree to which the social mobile app users would like to engage in e-WOM regarding consumption-related experience. In this way, e-WOM intention was operationalised in the previous studies following the suggestion of Venkatesh et al., [132]. To demonstrate, Mishra et al. [133], carried out a study adopting a similar sort of definition. This phenomenon has the potential to enhance sales and brand images among consumers [134]. The significance of e-WOM was unearthed in many studies in the marketing domain. For example, several studies have been carried out to uncover the influence of e-WOM on mobile banking adoption [135], purchasing intention [136], brand image, brand loyalty, purchase intent, brand awareness [137,138], repurchase intention [139], and customer satisfaction [140]. Further, a great deal of studies also focused on the motives and antecedents of e-WOM [[141], [142], [143], [144], [145], [146], [147], [148]]. These studies highlight the importance of e-WOM in consumer behaviour.

### 2.2 Hypotheses development

#### 2.2.1 Extraversion and self-presentation

Social media users usually involve in online self-presentation [149]. Specifically, individuals with extraversion traits are generally concerned about the impact of their image and behaviour [150]. And, they are comfortable with social situations [151] and these individuals have high value for social media [20]. For this reason, extraversion has been observed significantly associated with the desire for status [152]. To achieve this status, social media offer a variety of unique platforms for users to project their self-presentation [153]. Supporting them, Lee and Borah [7] stated that social media helps in building the self-presentation of users by offering simulation-like real-life communication. Considering this, past studies have embraced self-presentation as an impression [83,84]. Thus, the influence of extraversion had an effect on self-enhancement [15] and self-presentation [154]. On the basis of this discussion, we hypothesise that.

H1 Extraversion has a favourable influence on self-presentation.

#### 2.2.2 Extraversion and opinion leadership

Individuals with extraversion traits invite others’ attention and interest, welcome them for social interaction, and are highly connected to others on social media [111]. Note that they are very active on SNS [155]. These individuals with extraversion traits are known for being socially skilful, interpersonal, social linkages, and eager to take on activities [156] and like to be known by others [157]. Supporting them Akdevelioglu and Kara [111] highlighted the role of extraversion in identifying opinion leadership. Past investigations revealed that extraversion had a direct impact on opinion leadership [158], general opinion leadership [159], and social media opinion leadership [111]. On the backdrop of this reasoning, we posit that.

H2 Extraversion has a positive influence on opinion leadership.

#### 2.2.3 Extraversion and customer engagement

Customer engagement is seen as customers' resource investment in interactions with brands [160]. The intensity of customer participation in organizational activities or offering that organization or customer initiates [123] may depend on the personality traits of individuals. Since extraverts are very attentive to others and comfortable in social situations [151], these individuals are ready to engage, participate and express their feelings in group gatherings [91,153]. As a result, extroverts have a high propensity to use social media for communication purposes [111]. For such reasons, they have high propensity to interact in social media settings. Associating them, studies also unearthed the influence of extraversion on consumer participation [161], involvement in online brand community [162], customer engagement and online customer engagement [73,163,164]. Thus, the personality-engagement process warrants researchers' attention to discern individuals’ engagement phenomenon [163]. Based on this literature support, we posit that.

H3 Extraversion positively influences customer engagement.

#### 2.2.4 Extraversion and self-expression

Extroverts are the one who reflect their personalities by enhancing more connection [165] because these Individuals are sociable, outgoing, assertive, active, optimistic, chatty, lively, conversational and dynamic [166, 167]. And these individuals have the willingness to express their hedonism and symbolism nature through their consumption experiences as they have a high level of confidence, enthusiasm, assertiveness etc., [168]. These individuals use social media to communicate with others [21] as it offers enormous opportunities for actively engaging with brands to share their experiences. And, they are more inclined to enjoy sharing their thoughts and opinion with others as they have high propensity to articulate. Further, Wallace et al. [169], found that liking is also an expression of involvement with brands that support the self-concept. Customers who have developed a positive affect with the brand are generally prompted to express. Consequently, the influence of personality traits on self-disclosure and self-expression was observed widely [91,170]. Therefore, on the basis of these reviews, we hypothesise that.

H4 Extraversion has a positive influence on self-expression.

#### 2.2.5 Materialism and self-presentation

Materialism is an important individual difference which is greatly connected to different motives [171]. Usually, it is defined as “the importance consumers attach to worldly possessions” [97]. Materialistic people are willing to use products to convey information about themselves, signalling identity and status to others. Thus, materialistic consumers incline to engage in conspicuous consumption buying goods and displaying wealth publicly to sustain or achieve social status [172]. Further, “Self-presentation includes all strategies individuals use to establish an identity for the community audience in an online context” [173]. According to the situational cues, individuals manage their self-presentation based on their psychological motive for conspicuousness [174]. This motive can be an unconscious expression of self and symbolic expression using the brand to build and maintain an individual identity [175]. Moreover, self-presentation was also explored through sharing information on luxury consumption in social media [176]. Therefore, materialism had a significant impact on self-monitoring [177]. Following this logic, we postulate that.

H5 Materialism has a favourable effect on self-presentation.

#### 2.2.6 Materialism and self-expression

Materialism and wealth possessions is a symbol of success in life [178]. Thus, materialism is viewed as the inclination to lead materialistic life and the crucial way to aspire for end states and achieve life goals [96]. This materialistic personality may think that it is not possible to attain social status without attaining enough possessions [100]. Indeed, a past study showed materialistic customers are willing to use products to covey themselves to others signalling identity or status [27]. Supporting them, evidence from a recent study applying self-enhancement motive and impression management framework revealed that materialism plays a vital role in determining shopping values and purchase intention [179]. In order to attract attention and express themselves, these individuals are involved in the consumption of branded products [180]. If individuals seek to differentiate themselves from others when they express themselves in public settings, they are supposed to disclose their consumption activities to reveal themselves [181]. Thus, we posit that.

H6 Materialism has a positive influence on self-expression.

#### 2.2.7 Self-presentation and e-WOM

In order to reflect on their self-concept, people indulge in sharing and disclosing information online. Individuals show positive behavioural intentions toward a brand and product when they are content with the product [182]. Consequently, these individuals may post their consumption experiences on social media. Higher the propensity level of self-presentation, the better the number of content contributions in online forums [34]. As a result, self-promoting motives on social media are mostly affected by hedonic acquisitions [183]. When consumers attained an idealized self-image with the brand from their consumption experience, they express positive behavioural intention [184]. Sharing information about film, music and fitness app uses on social media self-presentation can be achieved [74,185]. Following this, previous studies disclosed the effect of self-presentation on e-WOM behaviour on SNS [15,85]. Based on this discussion, we expect that.

H7 Self-presentation has a positive impact on attitude towards e-WOM.

#### 2.2.8 Opinion leadership and e-WOM

Opinion leadership means the degree to which a person is seen as a model for others and the extent to which the information offered by him is considered interesting [50]. Further, opinion leadership is extrinsic, self-oriented and it is recognised as a major motivational driver for creating content in online communities [34]. By sharing information on a brand or product, one can express the self-concept and improve social status [181]. More content is contributed to social media in order to enhance their self-image if they have a higher level of opinion leadership motivation [186]. Thus, opinion leadership enhance the propensity of individuals to communicate with a larger audience. Supporting this, studies found that opinion leadership motivation had a positive influence on e-WOM behaviour [11,187]. Thus, we hypothesise that.

H8 Opinion leadership has a favourable influence on e-WOM.

#### 2.2.9 Customer engagement and e-WOM

Customer engagement is also seen as voluntary interaction with firms and contributing to business houses’ marketing activities beyond financial aspects [59] to meet certain levels of emotional, behavioural and cognitive activity with brands [120] or product review which is shared on social media. This type of customer engagement is facilitated by digital platforms [188] and social media is one way to attain this [54]. Following this concept, previous studies uncovered that customer engagement had a positive influence on interaction with virtual communities [189] customer participation [190], word of mouth [191,192], advocating the brands, giving and passing e-WOMs [193,194]. Accordingly, we hypothesise that.

H9 Extraversion favourably affects customer engagement.

#### 2.2.10 Self-expression and e-WOM

Self-expression is built on the general theme that consumers may recognise the satiation after their needs have been met when they gratify their needs, and this self-expression signifies the consumers’ preference for signalling identity through consuming customised products [195]. When consumers use the brand with the intent of self-expression, the connection between brand representation and self-representation becomes stronger [196]. This may enhance self-enhancement as customers discuss their favourite brand with others to improve self-expression. Further, a study also revealed that self-expression leads to disclosure and participation on social media [197]. The reviews highlight that self-expression is not just about interaction or engagement with the brand but also disclosing the information among the members. Based on this argument, we hypothesise that.

H10 Self-expression is positively related to attitude towards e-WOM

#### 2.2.11 Attitude towards e-WOM and intent to e-WOM

Attitude towards e-WOM are referred to here as a feeling of “goodness or badness” about e-WOM communication. Briefly, “person's evaluation of an object on a favourable to unfavourable continuum” [198]. As a result, the attitude has a major impact on individuals' intention to perform a specific behaviour [127]. This relationship is commonly applied in consumer behaviour studies adopting a theory of planned behaviour. Following this theory, it is expected that when the attitude is positive, its impact on intention may be stronger. Adopting this framework a number of studies took place to appreciate consumer behaviour [[199], [200], [201]]. Thus, the e-WOM attitude has a favourable influence on intent to e-WOM on social media [202]. Thus, we anticipate that.

H11 Social mobile app users' attitude towards e-WOM is positively related to intent to e-WOM.

#### 2.2.12 Moderating role of psychological impact

Psychological distress is referred to as the reaction of people to internal as well as external stresses and it is designated by a combination of anxiety, poor self-esteem, hopelessness, sadness and confused thinking [203]. These psychiatric symptoms due to the prevalence of the COVID-19 pandemic contributed to the psychological impact on people [204]. In order to account for the influence of psychological impact, this investigation adopted sub-constructs such as Covid-19 fear, traumatic stress, loneliness, and cabin fever syndrome [205]. When the WHO first announced a global public health emergency during the initial stage of COVID-19, most people were gripped by fear of uncertainty and the unknown. Specifically, this phenomenon is true as so much speculation around made on the rate of transmission that the disease would spread with no definitive treatment available in the market presently. Individuals started to feel cave syndrome when they have to go for self-isolation and quarantine occasionally. Consequently, people could not involve in social exchanges like meeting friends and relatives which led to the emergence of mental illness. In order to improve the mental health, and alleviate anxiety and stress, people started to use technology amid of pandemic [206]. Specifically, when they are quarantined, sharing COVID-related information is negatively related to psychological well-being whereas disclosing non-COVID information is connected to positive psychological well-being [207]. Further, everyone experienced a certain level of loneliness during the pandemic. Loneliness is “the unpleasant experience that occurs when a person's network of social relations is deficient in some important way” [208]. Interestingly, quarantines and voluntary social distancing are favourably associated with social media usage during the COVID-19 crisis [205]. Consequently, social media is associated with distress [209]. This gave enormous opportunities for social mobile users to disclose information in pursuit of social exchange. During the pandemic, the consumption and disclosure of information played a vital role to reduce the pandemic's impact [210]. With constrained resources for socialization, consumption and sharing of information are two major factors that contribute to social exchange for minimising the psychological impact through social mobile apps. Based on this literature support and reasoning, the formulated hypotheses are.

H12a The influence of self-presentation on the propensity to e-WOM is greater for social mobile app users with high psychological impact than less psychological impact.

H12b The influence of opinion leadership on the propensity to e-WOM is greater for social mobile app users with high psychological impact than less psychological impact.

H12c The influence of customer engagement on the propensity to e-WOM is greater for social mobile app users with high psychological impact than less psychological impact.

H12d The influence of self-expression on the propensity to e-WOM is greater for social mobile app users with high psychological impact than less psychological impact.

## 3 Methodology

### 3.1 Data collection and survey administration

This study relied on an online survey method and convenience sample to examine the research hypotheses. We recruited 627 social mobile app users in India. The respondent pool comprised 258 females (41%) and males (59%) from 16 to 52 years old. Before participating in the study, all respondents were asked to sign an informed consent form and the purpose of the study was also mentioned. Ethical approval was granted by the Doctoral scrutiny committee of “The Department of management studies, Indian Institute of Technology (ISM) Dhanbad”. For moderation (Multi-group) analysis, psychological impact measures were classified into low psychological impact and high psychological impact group. In order to test the gathered data, we examined the validity of the measurements and carried out a structural equation model.

### 3.2 Measures

The items adopted in this study were taken from previous research in the marketing domain and modified according the context. The questions were framed with a seven-point Likert scale ranging from 1 to 7. Whereby 1 is meant as totally disagree, 7 is referred to totally agree for all items. Items availed in this research are shown in Table 1.

**Table 1:** Measures and descriptive Statistics for the Constructs.

Measurement items	Loadings	CR	Alpha	AVE
**Extraversion** [211].		0.922	0.921	0.704
1. “I talk to a lot of different people using social mobile app”. 2. “I feel comfortable around people on social mobile app”. 3. “I start conversations using social mobile app”. 4. “I make friends easily on social mobile app”. 5. “I do not mind being the center of attention on social mobile app”.	0.82 0.86 0.87 0.84 0.80			
**Materialism** [213].		0.907	0.907	0.662
1. “I admire people who own expensive things”. 2. “The things I own say a lot about how well I'm doing in life”. 3. “I like to own nice things more than other people”. 4. “My possessions are important for my happiness”. 5. “I enjoy owning luxurious things”.	0.81 0.81 0.79 0.80 0.85			
**Self-presentation** [214]		0.884	0.881	0.656
1. “I like putting information about myself on social mobile apps”. 2. “I spend a considerable amount of time updating on social mobile apps about myself”. 3. “I use social mobile apps to promote myself”. 4. “I am very concerned about my social media appearance”	0.83 0.86 0.83 0.71			
**Opinion leadership** [109]		0.886	0.885	0.660
1. “I often persuade others to choose a particular brand that I like on social mobile apps” 2. “I often persuade others to agree with my opinion concerning”. 3. “I often influence others concerning the choice of a product”. 4. “I often influence others with my opinion on social mobile app”.	0.76 0.86 0.81 0.82			
**Self-expression** [181]		0.916	0.914	0.733
1. “I like to talk about what products and services I use on social mobile apps so people can get to know me better”. 2. “I like the attention I get when I talk to people on social mobile apps about the products and services I use”. 3. “I like the idea that people want to learn more about me through the products and services I use”. 4. “I like it when people pay attention to what I say on social mobile apps about my consumption activities”.	0.89 0.89 0.85 0.75			
**Customer engagement** [216].		0.898	0.905	0.639
1. “I would read posts related to brands on social mobile apps”. 2. “I would watch pictures related to brands on social mobile apps”. 3. “I would view videos related to brands on social mobile apps”. 4. “I would read comments related to brands on social mobile apps”. 5. “I would read fan pages related to brands on social mobile apps”.	0.83 0.84 0.77 0.80 0.79			
**Attitude towards e-WOM** [217]		0.931	0.931	0.819
1. “In general, e-WOM are a good thing.” 2. “In general, I like receiving e-WOM on social mobile app”. 3. “In general, e-WOM on social mobile app are a positive thing”.	0.82 0.88 0.90			
**e-WOM intention** [118]		0.906	0.905	0.764
1. “I intend to post my positive [negative] buying experience on social mobile apps”. 2. “I will try to post my positive [negative] buying experience on social mobile apps”. 3. “I plan to post my positive [negative] buying experience on social mobile apps”.	0.91 0.90 0.86			

#### 3.2.1 Extraversion

This investigation saw no objective in developing a new scale for measuring extraversion as the previously developed items are most suitable for present situation also. Trait extraversion was measured through the inventory of items developed by Goldberg, [211]. This measure was validated and applied in past studies by Islam et al. [73], Yoo et al. [212], and many other researchers. These items were used widely by researchers in internet and social media-related research, rewording them according to the context.

#### 3.2.2 Materialism

The short version of the trait materialism items was proposed and validated by Richins and Dawson [96]. Following this, Mowen and Spears [213] availed similar scale to measure compulsive buying of college students. Very recently, using a similar type of scale Moisescu et al. [30], evaluated the materialistic trait of social networking site users in their study to test a comprehensive framework. Since the scale should also be sufficiently objective to deliver similar outcome for different researchers, we had taken and framed along the line of Moisescu et al.,‘s [30] scale to measure social mobile app users' materialism.

#### 3.2.3 Self-presentation

There are scales to measure the self-presentation motives with reference to using SNS. Yet, this research exclusively borrowed the measures developed and evaluated by Kim et al. [214], as it is short, convenient, and validated by the same authors previously. This scale was later operationalised by Teichmann et al. [34], reframing and modifying depend on the context to investigate drivers of consumer-generated content in a model.

#### 3.2.4 Opinion leadership

In order to measure variable opinion leadership, the researcher availed the scale developed and tested by Flynn et al., [109]. Recently, modifying the original version, five-item scale was used by Chai et al. [11], to measure opinion leadership.

#### 3.2.5 Self-expression

In order to measure self-expression through consumption of products and service experience, 6 items scale were borrowed from Saenger et al., [181]. The same authors have also further empirically validated the scales. Plus, this scale was adopted very recently by Jung et al. [12], in their study.

#### 3.2.6 Customer engagement

Researchers had no consensus on the customer engagement scale or definition to analyse this construct [59]. With the purpose to bring out the respondents’ customer engagement on social media, this investigation used a scale developed by Muntinga [215]. This measures were later tested and validated by Tsai and Men [216] in their research. Because these items are designed to elicit the engagement of social media users at the intrinsic level, we had also adopted the same scale to explore the motives for customer engagement.

#### 3.2.7 Attitude towards e-WOM

Measuring attitude towards e-WOM was done with the help of items developed by Pollay and Mittal [217]. Originally, these authors used this scale to measure attitude towards advertising. As the communication on the internet platform started to emerge over the period time, researchers begun to apply the similar scale to understand e-WOM communication. This scale closely parallels Gvili and Levy's [218] notion of this construct. Later study ensured the items were validated, and this research framed and modified according to the social media context.

#### 3.2.8 e-WOM intention

e-WOM intention was rated using a scale developed by Ajzen [118]. Subsequently, there were many studies took place following similar items. Specifically, measures were validated and adopted by Fu et al. [202], and further modifications were made by Gruen et al. [219], Choi et al. [220], and Chu and Chen [221] in their studies according to the situation.

### 3.3 Group check

This study measured the psychological impact construct by borrowing 18 items (Cronbach's α = 0.894) from Chakraborty et al., [205]. This construct is a combination of sub-variables such as loneliness, COVID-19 fear, Traumatic stress, and Cabin fever syndrome. The summed average of items of psychological impact was computed for every respondents and then the sample was split into high and low groups using mean value 4.8 as the criteria point [222]. Whereas the low psychological impact (n = 289) group had a mean value of 3.931, the high psychological impact individuals (n = 307) had a mean value of 5.53. To make the analysis more accurate, this research not considered the respondents at the mean value (n = 31].

## 4 Data analysis

The data was examined following a two-stage method as described by Anderson and Gerbing [223]. First, CFA was performed, and subsequently the structural relationship was validated among variables. This research applied covariance-based SEM (CB-SEM).

### 4.1 Confirmatory factor analysis (CFA)

In order to test the model's goodness-of-fit, validity and reliability, CFA was carried out. The investigators followed similar procedures adopted in the recent studies [224,225]. The whole sample reveals a good overall fit of the model. Parsimony fit χ^2^/df = 2.686, CFI = 0.949, NFI = 0.922, IFI = 0.950, RFI = 0.912, TLI = 0.943, AGFI = 0.852, PGFI = 0.730, RMR = 0.094, RMSEA = 0.052. Next, the adequacy of the model was analysed through reliability and convergent validity tests which are exhibited in Table 1. AVE for each construct exceeded the minimum threshold of 0.5: extraversion = 0.704, materialism = 0.662, self-presentation = 0.656, opinion leadership = 0.660, self-expression = 0.733, customer engagement = 0.639, attitude towards e-WOM = 0.819, e-WOM intention = 0.764. Further, factor loadings of items belongs to constructs such as extraversion, materialism, self-presentation, opinion leadership, self-expression, customer engagement, attitude towards e-WOM, e-WOM intention ranged between 0.7 and 0.9, except for an item in self-presentation which was removed. Interestingly, high numbers were observed for attitude towards e-WOM and e-WOM intention. Thus, convergent validity was ensured [226,227]. The outcomes are presented in Table 1. This attests to the fact that measurement items have internal consistency and high reliability. Moreover, CR for the each construct is greater than 0.8: extraversion = 0.922, materialism = 0.907, self-presentation = 0.884, opinion leadership = 0.886, self-expression = 0.916, customer engagement = 0.898, attitude towards e-WOM = 0.931, e-WOM intention = 0.906. Thus, CR confirmed favourable (Table 1). As displayed in Table (2), the criteria for discriminant validity were met, the square root of AVE for each construct was found to be higher than the correlation values in the matrix [228]. In yet another way, discriminant validity is also attained when MSV is lower than the AVE as recommended by Hair et al., [229]. According to the analysis, all the MSV values are lower than AVE as Table 1, Table 2 exhibit. Therefore, discriminant validity is confirmed ultimately.

**Table 2:** Reliability and validity measures.

	MSV	MaxR [H]	MAT	EXT	CE	SE	OL	SP	IWOM	AWOM
MAT	0.368	0.909	0.813							
EXT	0.405	0.924	0.412	0.839						
CE	0.368	0.922	0.607	0.580	0.800					
SE	0.391	0.925	0.460	0.492	0.474	0.856				
OL	0.243	0.890	0.386	0.387	0.324	0.409	0.813			
SP	0.002	0.894	−0.005	0.012	0.045	0.047	−0.036	0.810		
IWOM	0.308	0.912	0.432	0.411	0.351	0.412	0.344	−0.026	0.874	
AWOM	0.405	0.934	0.600	0.637	0.560	0.625	0.493	−0.001	0.555	0.905

Note: *p⩽0.05 ***p⩽ 0.001.

### 4.2 Structural model assessment, hypotheses testing and results

**Fig. 1:**
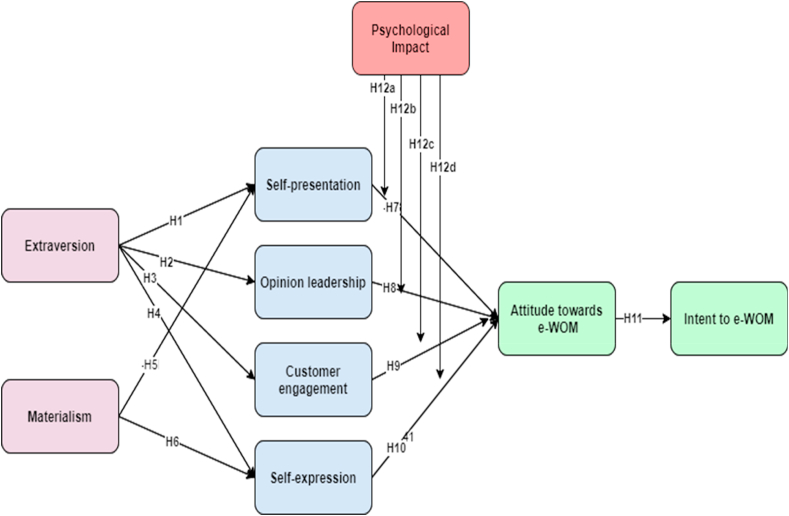
Hypothesised framework of the study.

We subsequently assessed the structural relationships (Fig. 1) using AMOS 21 among the latent variables after testing the measurement model. The analysis confirmed the proposed comprehensive model is an approximate representation of the underlying data. The overall goodness-of-fit for the whole sample structural model was acceptable given that the sample size is greater than 500 (229); x^2^/df = 3.460, CFI = 0.924, NFI = 0.896, RFI = 0.887, IFI = 0.924, TLI = 0.917, GFI = 0.855, PGFI = 0.737, AGFI = 0.832, and RMSEA = 0.063. As per Hair et al. [229], guidelines, if the value of coefficient determination (R^2^) is higher than 0.2, the model is considered acceptable. In this research, R^2^ for social media users’ attitude towards e-WOM is 0.49 and for intention to e-WOM is 0.29. Further, intrinsic motives customer engagement and self-expression also attained the significance with values 0.40 and 0.28 respectively. However, extrinsic motives self-presentation and opinion leadership have not shown statistical significance as they have an adverse results in the model.

**Fig. 2:**
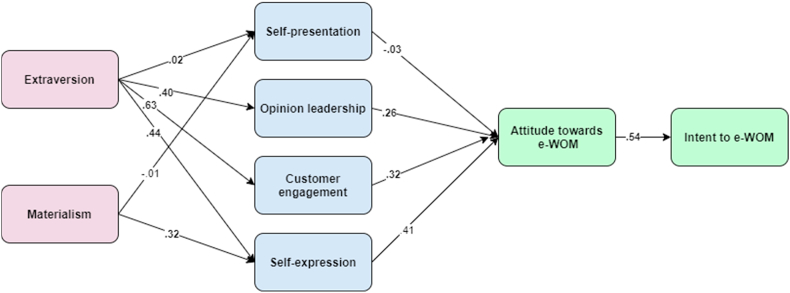
Hypothesises analysis results.

The comprehensive results of the hypotheses test and standardised path coefficients are shown in Figure (2) and Table (3) which display that extraversion had a significant influence on opinion leadership (β = 0.40, p < 0.001), customer engagement (β = 0.63, p < 0.001) and self-expression (β = 0.44, p < 0.001]. Next, the influence of materialism on self-expression (β = 0.32, p < 0.001) was also observed. Yet, the outcome showed that the influence of extraversion (β = 0.02, p > 0.05) and materialism (β = −0.01, p > 0.05) had no significant impact on self-presentation. Thus, H2-H4 & H6 are supported; yet H1 & H5 are not accepted.

**Table 3:** Hypothesises analysis results [standardised regression weights].

Hypotheses		Estimate	Results
H1	EXT→SP	.02	**Rejected**
H2	EXT→OL	.39***	Accepted
H3	EXT→CE	.63***	Accepted
H4	EXT →SE	.44***	Accepted
H5	MAT→SP	−.01	**Rejected**
H6	MAT→SE	.32***	Accepted
H7	SP→ Ae-WOM	−.03	**Rejected**
H8	OL→ Ae-WOM	.26***	Accepted
H9	CE→ Ae-WOM	.32***	Accepted
H10	SE→ Ae-WOM	.41***	Accepted
H11	Ae-WOM→ Ie-WOM	.54***	Accepted

*p < 0.05. **p < 0.01. ***p < 0.001.

Noticeably, the personality traits constructs which include extraversion (H1) and materialism (H2) had been observed not influencing self-presentation significantly. Arguably, the covid-19 pandemic made individuals exchange information on virtual platforms in different situations [230]. However, individuals with extroversion used social media with the motivation of passing time and maintaining relationships [231]. Adding further, extraversion is mostly linked to using video chat each day during the pandemic [230]. And, extraversion is also associated with well-being and loneliness when the curfew was imposed during the pandemic. Likewise, research also revealed that a higher level of materialism was highly related to attitude towards anxiety and lockdown [232]. Though materialism and extraversion had an impact on the usage of social mobile applications [233], people used social media to involve themselves to disclose information and stay connected during the Covid-19 pandemic. Due to these reasons, personality traits materialism, and extraversion may not have influenced social mobile app users’ self-presentation motive.

Further, the influence of opinion leadership (β = 0.26, p < 0.001), customer engagement (β = 0.32, p < 0.001), and self-expression (β = 0.41, p < 0.001) on attitude towards e-WOM were positive and significant, whereas the effect of self-presentation on attitude towards e-WOM was negative (β = −0.03, p > 0.05). Finally, the result reveals that attitudes toward e-WOM had a positive relationship with intent to e-WOM (β = 0.54, p < 0.001). In this way, H8, H9, H10, and H11 are supported but H7 is rejected.

There is not sufficient work to prove why the self-presentation (H7) had no impact on e-WOM during the pandemic. This could be due to the severity of the Covid-19 pandemic. Individuals shared pandemic-related situations, anxiety, boredom, and some other positive version by projecting home on social media during the pandemic. Briefly, social problems, adaptation to new lifestyles, and self-expression were recognised as major issues disclosed during the time of the Covid-19 pandemic [234]. They also argued that people relied on social media for self-presentation during the period of self-isolation as there is the unavailability of traditional communication. Yet, social mobile app usage was mostly associated with well-being [235] and coping during the pandemic [236].

The moderator (Multi-group) analysis was performed to examine the role of psychological impact on attitude towards e-WOM, testing hypotheses 12a-12d (Table 4). The outcome of the multi-group analysis exhibits that the path coefficient relationships-self-presentation and attitude towards e-WOM, and opinion leadership and attitude towards e-WOM for low and high psychological impact groups have not shown sufficient differences statistically. Thus, hypothesis 12a & b is not accepted. However, the magnitude of the path coefficient value for the influence of customer engagement on attitude towards e-WOM for (High group = 0.463) is stronger than (Low group = 0.252). Thus, H12c is supported. Though there was a difference between the (High group = 0.434) and (Low group = 0.376) groups for the impact of self-expression on attitude towards e-WOM, the difference was not statistically significant. Therefore, H12d is rejected. As stated earlier the reason for rejection of hypotheses 12a, b & d may be due to the usage of social mobile app with social exchange motives than intrinsic-extrinsic motives during the pandemic.

**Table 4:** Path Coefficients for moderating Effect of psychological impact.

Hyp otheses	LOW [β]	SE	High [β]	SE	T value	p-value	Results
12a	−.039	−.058	−.034	.048	0.066	>0.05	**Rejected**
12b	.346	.070	.250	.054	1.085	>0.05	**Rejected**
12c	.252	.057	.463	.071	2.317	<0.001	Accepted
12d	.376	.058	.434	.051	0.750	>0.05	**Rejected**

**Fig. 3:**
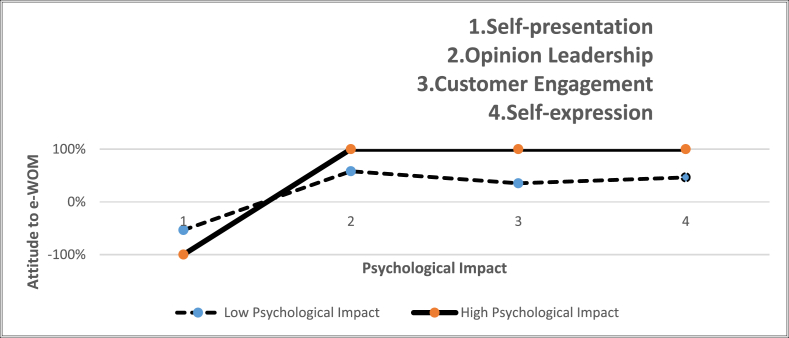
Graph indicating the differences and associations between low psychological impact and high psychological impact group.

The graph (Fig. 3) displays the concurrence of the influence of intrinsic and extrinsic motivational drivers on the propensity to e-WOM for low and high psychological impact groups “during the COVID-19 pandemic”.

## 5 Discussion

Drivers of e-WOM behaviour attracted the attention of many scholars and researchers in the recent past [30,32,72,237]. However, there is a lack of investigations on how personality traits affected e-WOM on SNS at the times of pandemic. To this end, our study is also in the lead to uncover the e-WOM behaviour of individuals considering psychological impact in a comprehensive framework.

The outcomes of this study reveal that when the relationship between customer engagement motive and attitude towards e-WOM is stronger, the individuals with high psychological impact seek to engage in e-WOM behaviour more than people with low psychological impact during the COVID-19 pandemic. We argue that people engage in e-WOM to relieve their stress, loneliness, fear and cabin fever syndrome. A study carried out by Matook et al. [238], proclaimed similar findings in past. Previous studies also brought out that how dark personality traits such as vulnerable narcissism, grandiose narcissism and psychopathy have played a significant role in predicting self-presentation on social media [45]. In contrary, our study states that personality traits failed to predict self-presentation on social media. Further, the effect of self-presentation on attitude towards e-WOM was also negative for people with high as well as low psychological impact groups, signifying that individuals may not involve in presenting themselves during the emergency situation with the intention to build their self-image. Further, the influence of opinion leadership on attitude towards e-WOM for high and low psychological impact groups had no significant difference, indicating that irrespective of the level of psychological impact, people are willing to share their thoughts on their consumption experience.

The impact of self-expression on the propensity to e-WOM behaviour was the highest among the motivational drivers, implying that during the pandemic also people are interested in expressing their self-image through consumption experience. Researching individuals’ brand-related engagements on SNS, de Vries et al. [67], found that expression motivation led to consumer-generated content online. This is because the brand has been used by consumers as a vehicle for self-expression to disclose the meaningful identity and symbolic value [239]. Similarly, a study by Choi and Burnham [240] also observed a higher relationship between self-expression and disclosing behaviour. Nevertheless, the construct-self-expression has been manifested many ways in different context like, Burgers et al. [241], and Walther [116] observed self-expression as the consequence of using of social media in their study, whereas in our study it is considered as a disclosure motive to express consumption of product experience. Further, other motivational drivers opinion leadership and customer engagement had a notable effect on attitude towards e-WOM or self-disclosure as studies explored in the past [11,187,197]. As discussed earlier, impact of self-presentation on attitude towards e-WOM was negative.

Extraversion had the strongest influence on customer engagement and self-expression in the model, suggesting that extroverts are closely associated with intrinsic motivation. This research also revealed the role of extraversion in predicting opinion leadership. Likewise, the study by Akdevelioglu and Kara [111] unearthed that the personality construct - extraversion has significant effect on social media opinion leadership. This is theoretically reassuring that higher the level of opinion leadership motivation, the greater the propensity to e-WOM on social media. Nonetheless, the previous study was carried out much before the pandemic, considering country-level differences as moderators. The present study is different employing intrinsic-extrinsic motivation along with personality traits to uncover the significance of e-WOM communication, applying psychological impact as a moderator. Further, Bilro and Loureiro [242] brought to light that intrinsic motivations favourably and significantly influenced utilitarian and hedonic rewards in studying customer engagement in online brand communities. They also disclosed that utilitarian and hedonic rewards were subsequently associated with brand advocacy in a comprehensive model. Similarly, our results also reflect that individuals with intrinsic motivations are more favour to participate in e-WOM.

Likewise, materialism has also been observed to be influencing self-expression favourably and self-presentation unfavourably. Realising the important role of extraversion and materialism in e-WOM, Moisescu et al. [30], used personality traits as drivers of e-WOM diffusion in a comprehensive approach. In addition, Tata et al. [32], developed an integrated model applying personality traits, and intrinsic-extrinsic motivational drivers in predicting e-WOM behaviour. Later, knowing the significance of consumers' behaviour during the pandemic, Mishra et al. [232], argued the moderating role of materialism on the association between anxiety and attitude towards consumption. Arguing in the same line, Moldes et al. [243], observed the increase in level of materialism during the pandemic owing to anxiety, stress, and excessive media consumption. However, intuitively we can argue that the stress and anxiety may discourage materialistic behaviour. On this line, this study is seemingly different from the past studies, and raison d'être is that it integrates psychological impact, materialism and extraversion traits collectively in the model to understand the influence of different dimensions of intrinsic and extrinsic motivational drivers on e-WOM behaviour. As noted earlier, this study is unique in bridging e-WOM communication with personality theory, and intrinsic and extrinsic motivational drivers considering the pandemic situation.

### 5.1 Theoretical contributions

This investigation contributes to marketing literature in many ways. Personality traits materialism and extraversion were not associated with self-presentation; nevertheless, these traits’ relationship with other motives like opinion leadership, customer engagement and self-expression were significant. These relationship highlights that social mobile app users may have other major motive like need for sharing information amid of pandemic. Hence, this comprehensive approach widens the scope of SET along the line of past studies undertaken in the social media domain [78]. Plus, the consequence of self-presentation on attitude towards e-WOM was also negative implying that social media users were not interested in self-enhancement amid the pandemic. Therefore, this investigation extends previous studies by offering a new perspective to e-WOM domain underpinning self-presentation theory. Besides, some recent work has associated self-presentation with psychological impact [105,244]. Although these researches brought out the determinants of self-presentation, encompassing trait theory and network size in the context of social media [15,245], the underlying reasons for intrinsic and extrinsic motivational drivers of e-WOM behaviour of social app users during COVID-19 pandemic are yet to be explored. Most of the previous investigations were centred on investigating the general consumer behaviour in social media. Further, the studies signifying the psychological impact and underlying motives towards e-WOM behaviour during the pandemic are sparse. Thus, the current research widens the prior knowledge by examining the role of motivational factors in e-WOM behaviour within the framework of social exchange, trait and self-presentation theories.

This study demonstrates a deeper understanding of the effect of psychological impact on the relationships between intrinsic-extrinsic motivational drivers, and e-WOM behaviour. But, the moderating role of psychological impact was not significant as expected. Interestingly, the difference between the impact of customer engagement on e-WOM for the high and low groups was significant. Further, the link between customer engagement and e-WOM behaviour is also favourable. Taken together, our model brings novel addition to the customer engagement concept embracing trait theory, and social exchange theory under a framework. Moreover, this study adds value to marketing literature, examining the significance of self-expression on the propensity to engage in e-WOM on social media at the time of pandemic underpinning uses and gratification theory (UGT) [246].

Furthermore, our findings strongly support the notion that opinion leadership motive is a critical factor in determining in e-WOM behaviour. Nonetheless, the consequence of opinion leadership on e-WOM behaviour did not differ significantly for the low and high psychological impact group. This work also confirmed that extraversion had a favourable effect on opinion leadership of social mobile app users to share consumption-related experiences. Hence, this investigation expands the horizon of theory of opinion leadership [247] by studying consequences and antecedents involving traits and behaviour in an integrated approach.

Although e-WOM in the social media domain is acknowledged, comprehensive analysis mapping e-WOM behaviour is scarce [248]. In more recent work, Moisescu et al. [30], urged researchers to explore personality traits along with other online characteristics to predict e-WOM. Responding to the gap, our research sheds light on e-WOM behaviour of social mobile app users, deploying the attitude-intention framework. As expected this work offers a significant contribution to the digital media domain under the purview of the social exchange theory and theory of planned behaviour. Hence, this research enlarges the scope of e-WOM domain.

A study found that loneliness, social unrest, psychological distress and social distancing played a significant role in SNS usage intensity during the pandemic [205]. Given this recent development, this study attempts to explore the relationship among psychological impact, personality traits and intrinsic-extrinsic motives in a comprehensive model, borrowing concepts from psychology, sociology and social psychology streams. Bridging different fields and applying a social psychological perspective, this study adds value to the existing e-WOM domain of knowledge. Notice that, the developed framework helps in appreciating the e-WOM behaviour of group of people during the pandemic. Thus, the current study expands the horizon of social psychology area in understanding human behaviour along the line of Chakraborty et al., [205].

### 5.2 Practical implications

This investigation offers several implications for social media managers, policymakers and marketing practitioners. From the study it was found that intrinsic motivational drivers had the strongest influence on attitude towards e-WOM in the model, suggesting that individuals with intrinsic motivational drivers are more into disclosing information and self-enhancement during the pandemic. Additionally, extrinsic motivation had a partial impact on the attitude towards e-WOM. Thus, marketers, policymakers and social media managers may reach to the people with intrinsic motivation to deliver the right information or review the product or situation. The results also assert that the relationship between extraversion and extrinsic motivation opinion leadership is stronger. Findings suggest that marketers may target social media users with opinion leadership motives and extraversion traits as they may be willing to review and comment on products they consumed during the pandemic. And these individuals can be a good brand ambassador for the brand they use. This implies that in some situation marketers can count on extrinsic motivational drivers in the segment to share the information.

Sharing is a major activity that takes place on social media and it is a vital element in understanding individuals’ self-presentation motive on the internet [249]. Yet, exploring the self-presentation motive of social mobile app users during the time of Covid-19 is unique due to the situation and the different kinds of lifestyle they maintain through the pandemic. The multi-group moderation analysis shows that there is not sufficient difference between the high and low levels of psychological impact group for examining the role of extrinsic motives self-presentation, opinion leadership in attitude towards e-WOM. This implies that irrespective of the level of psychological impact, people with extrinsic motives had a low propensity to e-WOM during the pandemic. Similarly, high and low levels of psychological impact group did not differ significantly for the relationship between self-expression and attitude towards e-WOM. Since the association between extrinsic motivations and attitude towards e-WOM is stronger, the takeaway for marketers is that individual may disclose information instinctively to share the information. Yet, they may not share information with a self-enhancement motive. Thus, it is not essential for marketers to give importance to firm-generated content with self-enhancement messages for the promotional campaign during the peak time of the pandemic. As stressed earlier, all the relationships which are associated with the self-presentation motive have not attained statistical significance in the model, signifying that customers are not willing to showcase and share experiences amid of COVID-19 pandemic with intention to show off. However, this research put forwards that social mobile app users may participate in interaction with others in order to alleviate their stress, loneliness, social distancing and anxiety during the pandemic. Therefore, policymakers are advised to consider the social engagement theory when they develop campaigns for the publicity of policies or services on social media.

Intrinsic motivations like self-expression and customer engagement had a remarkable effect on attitude towards e-WOM. According to the findings, generating customer engagement on social media, marketers can make individuals to e-WOM during the pandemic. This result would help brands to enhance their relationship with the customer in the short term. However, in order to develop a stronger e-WOM behaviour for consumption-related experiences, marketers may strive to develop a novel campaign, product and services to customers to engage them during the pandemic. Apart from business houses, governments can also employ engagement as a way to observe policies announced and new laws drafted before implementing the final one through social mobile app users’ participation. Specifically, people with customer engagement motives may be targeted to spread the right information during the COVID-19 pandemic.

Extroverts and materialistic people prefer to indulge in social media activities invariably. Recently, Moisescu et al. [30], announced that materialism had a significant relationship with e-WOM diffusion. Supporting them, the present study confirmed that extraversion made a remarkable impact on self-expression, customer engagement and opinion leadership. Though past researches have exposed elements of Big five traits as a key factor in determining e-WOM on social media, we recommend marketers target people with extraversion trait to get the message to the audience effectively and efficiently. Despite the prevalence of covid-19, consequences of materialism was observed on self-expression of consumption related experiences and its subsequent effect on e-WOM behaviour. Such findings reveal that business organisations still can produce and stock the products that are self-expressiveness in nature. Finally, framing an integrated model comprising materialism, extraversion and psychological impact, this research offers a comprehensive understanding and rich insights into intrinsic-extrinsic motivational drivers of e-WOM behaviour. Thus, we believe this study contributes to practitioners in business organisations and government by providing a sense of direction for their social media marketing campaigns.

## 6 Limitations and scope for future research

Though this investigation contributes to both the theoretical and practical aspects enormously, several limitations are associated with our research. This study measured traits, self-presentation and other motivations through self-reporting which can attract inaccuracies as respondents may have a biased response to questions related to socially desirable behaviour [7]. Since the study is carried out during the lockdown, most of the experience of consumers is based on the acquisition of materials and consumption. As a result, it failed to capture the nature of experiential services. Additionally, this investigation did not measure the intensity of extraversion and materialism classifying high or low among social mobile app users. This could be a potential research area for future investigations to study. The follow-up study may be carried out in different fields such as tourism, academic and professional achievements etc. Finally, other elements of the big five traits can also spur upcoming studies with a similar framework in different situation.

### 6.1 Conclusions

In sum, this research provides evidence that individuals with high psychological impact have a strong motivation to involve in customer engagement to participate in e-WOM communication. Nonetheless, there was no difference observed between high and low psychological impact groups for intrinsic-extrinsic motivational drivers such as self-expression, self-presentation and opinion leadership. Further, motivational drivers such as self-expression, opinion leadership and customer engagement had a notable effect on attitude towards e-WOM for the whole sample analysis. By contrast, self-presentation has influenced e-WOM behaviour negatively during the pandemic. Similarly, materialism had also affected self-presentation unfavourably. This represents a stark contrast from the past investigations. Maybe due to the prevalence of the COVID-19 pandemic, social mobile app users were more into the social exchange of information in order to relieve anxiety, loneliness, and other social issues. Although most researchers broadly agree that the impact of extraversion on self-presentation was observed to share information on social media, this investigation brought to light that the effect of extraversion on self-presentation is not significant. This implies that even the social mobile app users with extraversion traits may had been affected psychologically during the pandemic. However, trait extraversion significantly influenced opinion leadership, customer engagement and self-expression. Likewise, materialism had also formed a favourable impact on self-expression. And, attitude towards e-WOM strongly influenced intent to e-WOM. We conclude by stressing that this study offers interesting insights to practitioners and researchers in social media marketing and consumer behaviour domain.

## 7 Ethics statement

“All research phases were reviewed and approved by Doctoral scrutiny committee of Department of management studies, Indian Institute of Technology (ISM) Dhanbad which granted ethical approval. Consent from the respondents were obtained, and the participants’ identity remained anonymous”.

## 8 Informed consent statement

“Informed consent was obtained from all respondents participated in the study. In case of participants aged less than 18, Informed consent was obtained from their parents”.

## Author contribution statement

Balamoorthy S; Dr.Bibhas Chandra, PhD: Conceived and designed the experiments; Performed the experiments; Analyzed and interpreted the data; Contributed reagents, materials, analysis tools or data; Wrote the paper.

## Funding statement

This research did not receive any specific grant from funding agencies in the public, commercial, or not-for-profit sectors.

## Data availability statement

Due to the nature of this research, participants of this study did not agree for their data to be shared publicly, so supporting data is not available”.

## Declaration of interest’s statement

The authors declare no competing interests.
